# Impact of Werther Effect Exerted by Social Media on Individuals Presenting with Deliberate Self-Harm (DSH) to a Tertiary Care Hospital in Sri Lanka

**DOI:** 10.1192/bjo.2025.10247

**Published:** 2025-06-20

**Authors:** Isuri Wimalasiri, Aindralal Balasuriya, Madhubhashini Dayabandara

**Affiliations:** 1 General Sir John Kotelawela Defence University, Ratmalana, Sri Lanka; 2 Faculty of Medicine, University of Colombo, Colombo, Sri Lanka

## Abstract

**Aims:** There was an exponential increase in the rates of deliberate self-harm (DSH) in Sri Lanka over the last two decades coinciding with the rise of social media. Werther effect is the phenomenon where sensationalised media reports on suicide/DSH leads to increased rates of DSH/suicide. Therefore, assessing the impact of social media on DSH and implementing media regulations are timely needs. The objectives of this study included assessing the extent of exposure and the severity of emotional disturbance incurred due to social media content depicting DSH/suicide and determining the association between exposure and emulating DSH behaviour they were exposed to.

**Methods:** This descriptive cross-sectional study conducted in 2022, consisted of a sample of 162 individuals presenting with DSH to NHSL. A semi-structured clinical interview was used to collect data. Relevant statistical tests were used to analyse data.

**Results:** The average duration this study population spent on social media (1–3 hours) surpassed previous records for Sri Lankans (34 minutes). Fifty per cent were aged 20–29 years and 59.8% were female; a notable over-representation of minority religious and ethnic groups compared with general population demographics in Sri Lanka was seen. Out of 162 participants, 148 (91%) acknowledged being exposed to social media content depicting self-harm at some point. Facebook and YouTube were the top two platforms through which individuals were exposed to DSH/suicide content and the commonest methods published were hanging and overdose.

Although there was no statistically significant association between age and sharing self-harm content on social media, an overwhelming majority (87%) were <30 years of age. Approximately 30% of the sample reported experiencing severe emotional disturbance following exposure to DSH/suicide content.

Individuals who intentionally searched social media for DSH/suicide content consisted 11% of the sample and they were more likely to emulate similar behaviour than those who did not actively search such content. Spending 5 hours or more in a day on social media significantly increased several risks including the likelihood of being exposed to content depicting DSH/suicide, experiencing emotional disturbance and emulating the self-harm behaviours they were exposed to.

**Conclusion:** The notable ethnic and religious disparity seen in this sample suggests the need to examine healthcare accessibility, socioeconomic disparities, and tailored mental health literacy programmes. Prolonged social media exposure, particularly over 5 hours increases the risks of exposure and emulation significantly, warranting implementation of policies to reduce harmful content on social media and targeted interventions to promote healthy social media use among vulnerable populations.

